# Spontaneous Resolution of Radiotherapy-induced Craniopharyngioma Cyst

**DOI:** 10.7759/cureus.272

**Published:** 2015-05-28

**Authors:** Mario Teo, Fiona Cowie, Paul Fivey, Jerome St.George

**Affiliations:** 1 School of Medicine, Stanford University Medical Center; 2 Department of Paediatric Oncology, Royal Hospital for Sick Children, Glasgow, UK; 3 Department of Neurosurgery, Institute of Neurological Science, Glasgow, UK

**Keywords:** craniopharyngioma cyst, radiotherapy, spontaneous resolution

## Abstract

Craniopharyngioma cyst enlargement after surgery and radiation therapy is often presumed to represent a treatment failure, instigating further management strategies. We present an eight-year-old girl with a small intrasellar residuum post-resection who then developed cystic enlargement post-radiotherapy. With close surveillance, the cyst spontaneously resolved.

## Introduction

Craniopharyngioma cyst enlargement after surgery and radiation therapy is often presumed to represent a treatment failure, instigating further management strategies in the form of cyst aspiration, repeat surgery, instillation of cytotoxic agents, or further radiotherapy [1-6]. We present an eight-year-old girl with a small intrasellar residuum post-resection who then developed cystic enlargement post-radiotherapy.

Informed consent from her parents was obtained for treatment and publication. Details that might disclose the identity of the subject under study were omitted.

## Case presentation

**Figure 1:**
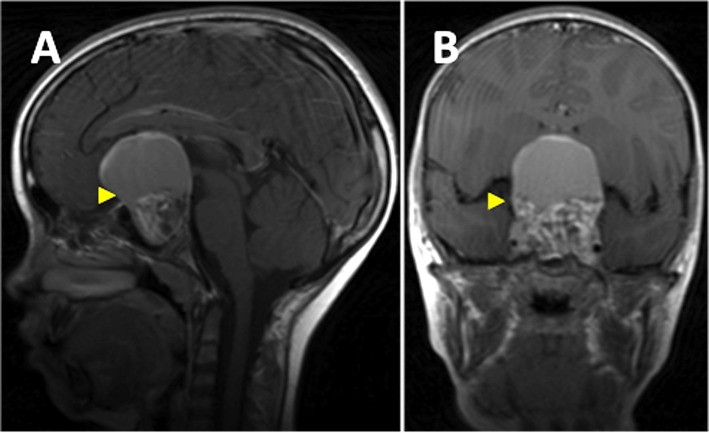
T1-weighted MRI brain with contrast (sagittal view [panel A], coronal view [panel B]) Shows a large partly cystic, partly solid craniopharyngioma (arrowhead) with suprasellar extension elevating the optic chiasm, third ventricle, and causing sellar expansion.

**Figure 2:**
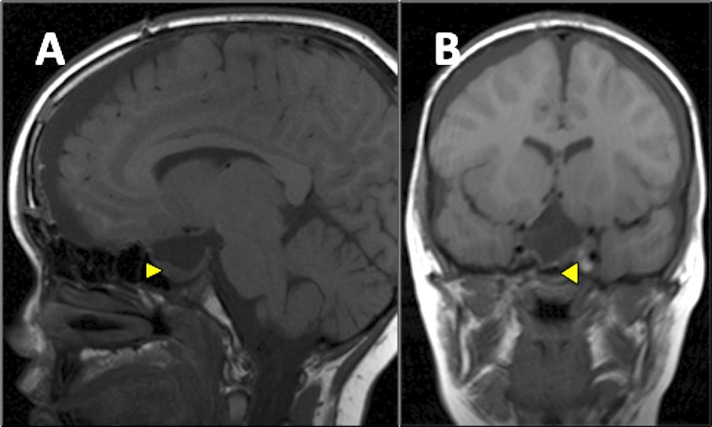
Postoperative T1-weighted MRI brain (sagittal view [panel A], coronal view [panel B]) Shows subtotal resection of the craniopharyngioma with only a small intrasellar residuum (arrowhead).

**Figure 3:**
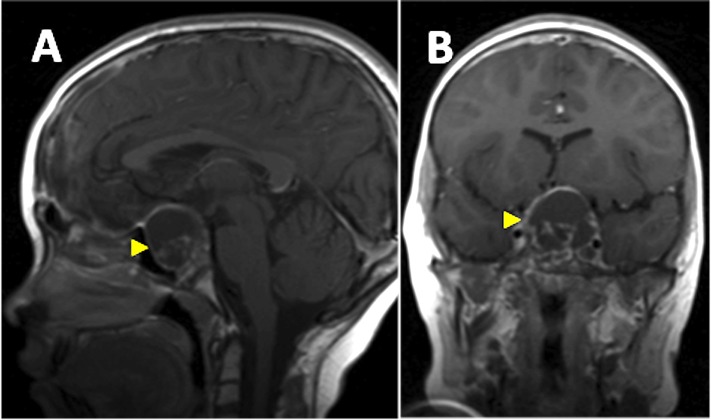
T1-weighted MRI brain with contrast (sagittal view [panel A], coronal view [panel B]) MRI performed post-radiotherapy when patient complained of recurrent dull headache, showing a recurrent cyst (arrowhead) with elevation and distortion of the third ventricle.

**Figure 4:**
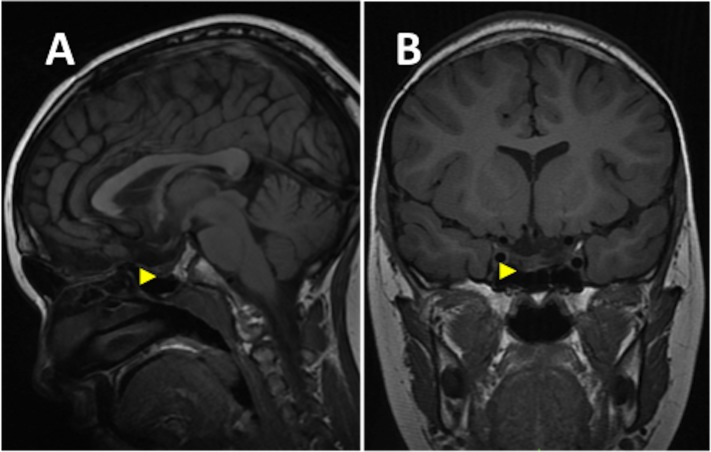
At 9 months post radiotherapy, T1-weighted FLAIR MRI brain (sagittal view [panel A], coronal view [panel B]) Shows resolution of the craniopharyngioma cyst leaving only the previously observed intrasellar residuum (arrowhead).

An eight-year-old girl presented with a one-year history of progressive headache and visual loss. MRI showed a large partly cystic, partly solid craniopharyngioma (Figure 1). A craniotomy and subtotal resection were performed with only an intrasellar residuum apparent on postoperative imaging (Figure 2). Her visual function improved and she subsequently received a six-week course of radiotherapy. Several weeks following that treatment, she complained of non-disabling recurrent dull headache, with no deterioration of her visual function. Repeat MRI showed a recurrent cyst (Figure 3). She was kept under close clinical, radiological and ophthalmological surveillance and nine months later, MRI revealed that the craniopharyngioma cyst had spontaneously resolved, leaving only the previously observed sellar residuum (Figure 4).

No tumour progression was noted at her seven year follow-up.

## Discussion

Craniopharyngiomas are benign slow-growing tumours located in the sellar and parasellar region and thought to originate from Rathke’s pouch [7-8].

Patients who demonstrate cyst enlargement after surgery and radiation therapy are often presumed to represent treatment failures [1-6]. Therapeutic approaches in various centers include repeat cyst aspirations, surgical re-excision, and installation of cytotoxic agents, such as bleomycin [1, 6, 9-10]. However, each intervention carries an associated morbidity. Surgical re-excision entails a higher risk of morbidity compared to the initial surgery [10]. Repeated cyst aspiration can be complicated by chemical meningitis. Further radiation increased the risk of optic neuropathy, brain necrosis, and slight long-term risk of malignancy [11-12]. In order to minimise the risk of treatment sequelae in the pediatric population, administration of radiotherapy with precision and conformality is important [12-14]. Although photon radiotherapy is regularly used to treat pediatric tumors, for complex volumes or volumes close to critical structures like the optic apparatus, protons provide better conformality [15]; therefore, it has been used in patients with craniopharyngioma.

A recent USA study included 24 pediatric patients with biopsy-proven craniopharyngioma who were treated with proton radiotherapy [6]. At the initial diagnosis, four patients had undergone gross total resection, 16 underwent subtotal resection, and four patients had cyst drainage with biopsy. Of the 17 children who underwent repeat imaging during radiotherapy, six required intervention because of changes in the cyst dimensions. Four patients (24%) had cyst growth beyond the original treatment fields, requiring enlargement of the treatment plan. One patient’s treatment field was reduced after a decrease in cyst size. Cyst drainage was performed in another patient to avoid enlargement of the treatment fields. With a median follow-up of 40.5 months, eight patients developed cystic recurrence and underwent repeat resection; two underwent surgical excision three times.

However, in another more rare phenomenon, as highlighted in this case, cysts may develop post-radiotherapy and, without intervention, subsequently decrease in size. Constine, et al. [1] also made a similar observation in 1980s, when four of 11 patients with craniopharyngioma treated with surgery followed by radiation therapy demonstrated post-irradiation enlargement of a cystic component. In three patients, the subsequent decrease in size of the cysts was observed without surgical intervention, and size stabilization was observed in another one patient.

This observation might result from delayed radiation-induced damage to the craniopharyngioma cells, thus allowing cyst formation to continue transiently. A similar phenomenon has been previously observed in cystic vestibular schwannoma post-Gamma Knife radiosurgery, where the cystic component can undergo transient expansion followed by sustained regression [16]. Spontaneous rupture of the craniopharyngioma cyst might also explain the shrinkage. Such an event has been previously reported to cause episodes of aseptic meningitis [17]; however, it is unlikely that the child had a spontaneous rupture of the cyst (as she did not have aseptic meningitis) to explain its spontaneous resolution.

Most series denote recurrence as either a return of symptoms or radiographic changes consistent with tumour or cyst growth. In these series, the mean interval to such recurrence is 2.5 to 4.6 years [5, 18-19], but at times has been noted to occur in the early months after irradiation, as in our case. Therefore, if such event occurs in the first few months after therapy and is unaccompanied by clinical deterioration, observation alone should be considered.

## Conclusions

This is an important and educational observation because craniopharyngioma cyst enlargement after surgery and radiation therapy is often presumed to represent a treatment failure, instigating further management strategies. We present an eight-year-old girl with a small intrasellar residuum post-resection who then developed cystic enlargement post-radiotherapy. With close surveillance, the cyst spontaneously resolved, thereby avoiding any further intervention that might have an associated morbidity.
